# Differential Expression of Growth Factor Receptors and Membrane-Bound Tumor Markers for Imaging in Male and Female Breast Cancer

**DOI:** 10.1371/journal.pone.0053353

**Published:** 2013-01-04

**Authors:** Jeroen F. Vermeulen, Robert Kornegoor, Elsken van der Wall, Petra van der Groep, Paul J. van Diest

**Affiliations:** 1 Department of Pathology, University Medical Center Utrecht, Utrecht, The Netherlands; 2 Division of Internal Medicine and Dermatology, University Medical Center Utrecht, Utrecht, The Netherlands; Northwestern University Feinberg School of Medicine, United States of America

## Abstract

**Introduction:**

Male breast cancer accounts for 0.5–1% of all breast cancers and is generally diagnosed at higher stage than female breast cancers and therefore might benefit from earlier detection and targeted therapy. Except for HER2 and EGFR, little is known about expression of growth factor receptors in male breast cancer. We therefore investigated expression profiles of growth factor receptors and membrane-bound tumor markers in male breast cancer and gynecomastia, in comparison with female breast cancer.

**Methods:**

Tissue microarrays containing 133 male breast cancer and 32 gynecomastia cases were stained by immunohistochemistry for a panel of membrane-bound targets and compared with data on 266 female breast cancers.

**Results:**

Growth factor receptors were variably expressed in 4.5% (MET) up to 38.5% (IGF1-R) of male breast cancers. Compared to female breast cancer, IGF1-R and carbonic anhydrase 12 (CAXII) were more frequently and CD44v6, MET and FGFR2 less frequently expressed in male breast cancer. Expression of EGFR, HER2, CAIX, and GLUT1 was not significantly different between male and female breast cancer. Further, 48.1% of male breast cancers expressed at least one and 18.0% expressed multiple growth factor receptors. Since individual membrane receptors are expressed in only half of male breast cancers, a panel of membrane markers will be required for molecular imaging strategies to reach sensitivity. A potential panel of markers for molecular imaging, consisting of EGFR, IGF1-R, FGFR2, CD44v6, CAXII, GLUT1, and CD44v6 was positive in 77% of male breast cancers, comparable to female breast cancers.

**Conclusions:**

Expression patterns of growth factor receptors and hypoxia membrane proteins in male breast cancer are different from female breast cancer. For molecular imaging strategies, a putative panel consisting of markers for EGFR, IGF1-R, FGFR2, GLUT1, CAXII, CD44v6 was positive in 77% of cases and might be considered for development of molecular tracers for male breast cancer.

## Introduction

Breast cancer in males is a rare disease, accounting for 0.5–1% of all breast cancer cases [1], [2]. Male breast cancer patients generally present at higher age than female breast cancer patients and at a higher stage including more frequently lymph node metastases [1], [3], [4]. Furthermore, molecular subtypes of male breast cancer are differently distributed than female breast cancer, the most predominant subtype in male being Luminal A followed by Luminal B. HER2-driven subtypes have not been observed [5]–[7]. Conflicting data exist whether triple negative/basal-like breast cancers occur in male breast cancer, but at least it is infrequent [6]–[8].

With regard to potential druggable targets, knowledge on the expression of individual tumor markers is limited and variable. Estrogen Receptor α (ERα) and progesterone receptor (PR) expression in male breast cancer is present in around 90% of patients [4], which makes them eligible for adjuvant therapy using tamoxifen and aromatase inhibitors. HER2 expression in male ranges between 0–45% of cases in different studies [5]–[7], [9]–[11], but current consensus in recent studies shows that HER2 expression in male breast cancer is seen in no more than 3–7% of cases. The epidermal growth factor receptor (EGFR) is the only other growth factor receptor for which expression data is available in male breast cancer, suggesting that EGFR is expressed in 12–76% of cases [6], [7], [9], [11], [12].

Nowadays, antibody-based molecular therapies have been developed for e.g. HER2 [13], [14] and EGFR [15], [16] and molecular therapies for other growth factor receptors are still investigational. In addition to being therapeutic targets, growth factor receptors might be useful for molecular imaging [17]–[19]. Molecular imaging using optical near-infrared fluorescent probes has advantages compared to mammography alone, because probes can be conjugated to antibodies, antibody fragments or peptides which increases the specificity of the signal [20]. Further, near-infrared fluorescently labeled antibodies can be used for image-guided surgery, thereby enhancing radical resection of breast cancer and lymph node metastases [21]–[23]. We recently described that in addition to growth factor receptors, hypoxia upregulated proteins (carbonic anhydase IX (CAIX) and XII (CAXII), and GLUT1) and CD44 variants might be useful for molecular imaging of female breast cancer [24]. Because fluorescently labeled antibodies and antibody-fragments are not easily internalized, ERα and PR are not considered for optical imaging strategies. Selection of potential antibody-based agents for detection and therapy of male breast cancer is labor intensive and costly. Furthermore, the expression of membrane markers in male breast cancer is unknown.

Since male breast cancer patients have more frequently lymph node metastases, molecular imaging may be of benefit for males to assess stage of disease and monitor disease progression or response to therapy. In the present study, we therefore investigated by immunohistochemistry the expression of growth factor receptors and membrane markers in male breast cancer and compared the results with those we observed in female breast cancer and gynecomastia, in order to find a panel of potential markers for therapy and molecular imaging of male breast cancer.

## Materials and Methods

### Patients

The origin and composition of the male breast cancer study population was described before [7]. Female breast cancer cases from 2003–2007 were derived from the archive of the Department of Pathology University Medical Center Utrecht, Utrecht, The Netherlands as described before [25]. The study population comprised 133 cases of male and 266 cases of female invasive breast cancer. For all cases haematoxylin and eosin (HE) slides were reviewed by two experienced observers (PJvD, RK) to confirm the diagnosis and to characterize the tumor. Histological grade was assessed according to the modified Bloom and Richardson score [26], and mitotic activity index (MAI) was assessed as described before [27]. Clinicopathological characteristics of all male and female breast cancer cases are shown in Table 1.

**Table 1:** Clinicopathological characteristics of male and female breast cancer.

		Male	Female	
Feature	Grouping	N (%)	N (%)	p-value
Age (years)	Mean	66	59	
	Range	32–89	28 to 88	**<0.001**
Histological type	Invasive ductal cancer	121 (91.0)	211 (79.3)	
	Invasive lobular cancer	3 (2.3)	25 (9.4)	
	Others	9 (6.8)	30 (11.3)	**0.008**
Tumor size (cm)	≤2	73 (54.9)	130 (48.9)	
	>2 and ≤5	54 (40.6)	113 (42.5)	
	>5	2 (1.5)	21 (7.9)	
	Not available	4 (3.0)	2 (0.7)	**0.030**
Histological grade	1	32 (24.1)	45 (16.9)	
	2	54 (40.6)	100 (37.6)	
	3	47 (35.3)	121 (45.5)	0.094
Lymph node status	Negative *	51 (38.3)	119 (44.7)	
	Positive **	60 (45.2)	132 (49.6)	
	Not available	22 (16.5)	15 (5.7)	0.797
Mitotic index #	≤12	76 (57.1)	132 (49.6)	
	≥13	57 (42.9)	134 (50.4)	0.156
ERα †	Negative	8 (6.0)	53 (19.9)	
	Positive	125 (94.0)	213 (80.1)	**<0.001**
PR †	Negative	43 (32.3)	89 (33.5)	
	Positive	90 (67.7)	177 (66.5)	0.802

# : per2 mm^2^;

* : negative = N0 or N0(i+);

** : positive = ≥N1mi (according to TNM 7^th^ edition, 2010);

† : positive = ≥10% nuclear staining.

In addition, 32 gynecomastia cases from 2000–2010 were retrieved from the archives of the Department of Pathology of the University Medical Center Utrecht. Original HE slides were reviewed by two observers (PJvD, RK) to confirm the diagnosis, and to subtype gynecomastia (florid, intermediate, fibrous) as described before [28]. Tissue microarrays were constructed as described by Kornegoor et al. [7], [28]. Since we are using archival pathology material which does not interfere with patient care and does not involve physical involvement of the patient, no ethical approval is required according to Dutch legislation [29]. Use of anonymous or coded left over material for scientific purposes is part of the standard treatment contract with patients and therefore informed consent procedure was not required according to our institutional medical ethical review board, this has also been described by van Diest [30].

### Immunohistochemistry

Immunohistochemistry was carried out on 4 µm thick sections for a panel of growth factor receptors. After deparaffination and rehydration, endogenous peroxidase activity was blocked for 15 min in a buffer solution pH5.8 containing 0.3% hydrogen peroxide. After antigen retrieval, i.e. boiling for 20 min in 10 mM citrate pH6.0 (for progesterone receptor (PR), Hepatocyte growth factor receptor (MET), Fibroblast growth factor receptor 2 (FGFR2), CD44v6, CAXII, carbonic anhydrase 9 (CAIX), and GLUT1), or Tris/EDTA pH9.0 (estrogen receptor α (ERα), HER2, and Insulin-like Growth Factor-1 receptor (IGF1-R)) or Prot K (0.15 mg/ml) for 5 min at room temperature (EGFR), a cooling off period of 30 min preceded the primary antibody incubation. ERα (clone ID5, DAKO, Glostrup Denmark) 1∶200; PR (clone PgR636, DAKO) 1∶100; HER2 (SP3, Neomarkers, Duiven, The Netherlands) 1∶100; IGF1-R (NB110-87052, Novus Biologicals, Cambridge, UK) 1∶400; FGFR2 (M01, clone 1G3, Abnova, Heidelberg, Germany) 1∶800; CD44v6 (clone VFF18, BMS125, Bender MedSystems, Austria) 1∶500; GLUT1 (A3536, DAKO) 1∶200; CAXII (HPA008773, Sigma Aldrich, Zwijndrecht, The Netherlands) 1∶200; CAIX (ab15086, Abcam, Cambridge, UK) 1∶1,000 were incubated for 1 h at room temperature. Primary antibodies against EGFR (clone 31G7, Zymed, Invitrogen) 1∶30; MET (18-2257, Zymed, Invitrogen) 1∶100 were incubated overnight at 4°C. All primary antibodies were diluted in PBS containing 1% BSA.

The signal was amplified using Brightvision poly-HRP anti-mouse, rabbit, rat (DPVO-HRP, Immunologic, Duiven, The Netherlands) or the Novolink kit (Leica, Rijswijk, The Netherlands) (in the case of EGFR) and developed with diaminobenzidine, followed by counterstaining with haematoxylin, dehydration in alcohol and mounting.

### Scoring of Immunohistochemistry

All stainings were compared to normal breast tissue (obtained from female patients that underwent mammoplasty) and scored as positive when a clear membrane staining (2+ or 3+) was observed, except for HER2 where only a score of 3+ was considered positive. All scoring was done by JFV, RK and PJvD who were blinded to patient characteristics and results of other stainings. Expression data for the female breast cancers and for the hypoxia proteins in male breast cancer were derived from our previous studies [24], [31].

### Statistics

Statistical analysis was performed using IBM SPSS Statistics version 18.0 (SPSS Inc., Chicago, IL, USA). Associations between categorical variables were examined using the Pearson’s Chi-square test and associations between continuous variables using Student’s T-test. Logistic regression was used to correct for differences in ERα expression, tumor size (≤2 cm vs. >2 cm), age (<60 years vs. ≥60 years), and histological type (ductal vs. lobular/other histological type) between male and female breast cancers. P-values <0.05 were considered to be statistically significant.

## Results

**Figure 1:**
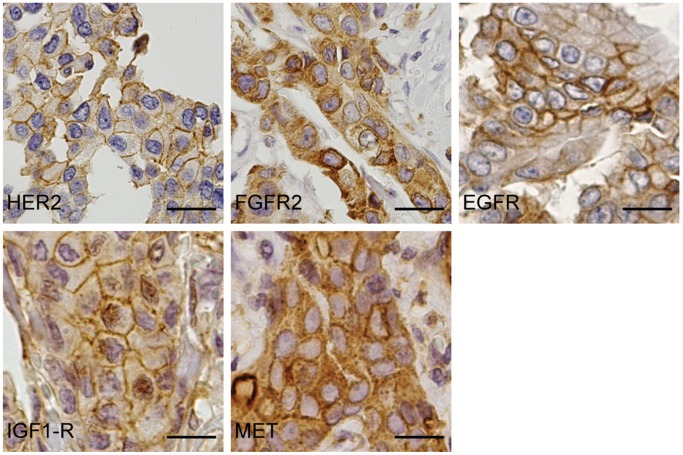
Expression of growth factor receptors in male breast cancer. Expression of HER2, FGFR2, EGFR, IGF1-R, and MET in representative cases of male breast cancer. Size bars equal 20 µm.

In our study population of 133 cases of male breast cancer, we found 4 cases (3.0%) expressing HER2, 15 cases (11.4%) EGFR, 6 cases (4.5%) MET, 16 cases (12.1%) FGFR2, and 50 cases (38.5%) IGF1-R (Table 2). Representative cases of male breast cancer are shown in Figure 1. CD44v6, CAIX, CAXII, and GLUT1 were expressed in 44 cases (33.3%), 9 cases (6.8%), 36 cases (27.1%), and 41 cases (31.3%) of male breast cancers, respectively.

**Table 2:** Expression of membrane markers in male and female invasive breast cancer.

Feature	Male	Female	Logistic regression*		
	N (%)	N (%)	p-value	OR	95% CI
IGF1-R					
Negative	80 (61.5)	219 (84.6)			
Positive	50 (38.5)	40 (15.4)	**<0.001**	3.317	1.948–5.647
HER2					
Negative	129 (97.0)	246 (92.5)			
Positive	4 (3.0)	20 (7.5)	0.316	0.555	0.175–1.754
EGFR					
Negative	117 (88.6)	226 (85.6)			
Positive	15 (11.4)	38 (14.4)	0.329	1.494	0.667–3.347
MET					
Negative	126 (95.5)	206 (78.0)			
Positive	6 (4.5)	58 (22.0)	**<0.001**	0.194	0.079–0.473
FGFR2					
Negative	116 (87.9)	195 (76.5)			
Positive	16 (12.1)	60 (23.5)	**0.001**	0.331	0.174–0.628
CD44v6					
Negative	88 (66.7)	93 (35.2)			
Positive	44 (33.3)	171 (64.8)	**<0.001**	0.269	0.168–0.432
GLUT1					
Negative	90 (68.7)	175 (72.6)			
Positive	41 (31.3)	66 (27.4)	0.164	1.439	0.862–2.401
CAIX					
Negative	123 (93.2)	215 (88.5)			
Positive	9 (6.8)	28 (11.5)	0.865	0.924	0.372–2.297
CAXII					
Negative	97 (72.9)	238 (90.8)			
Positive	36 (27.1)	24 (9.2)	**0.001**	2.753	1.501–5.049

* Correction for age, histology, ERα expression, and tumor size, Confidence Interval (CI), Odds Ratio (OR). OR >1 indicates higher expression in male.

### Membrane Protein Expression in Male Breast Cancer Compared to Female Breast Cancer

Compared to female breast cancer, IGF1-R was more frequently expressed in male breast cancer (p<0.001), while MET and FGFR2 were less frequently expressed (both p<0.001; Table 2) in male breast cancer. Expression of EGFR and HER2 was not significantly different between male and female breast cancer. Moreover, expression of any growth factor receptor was present in 64 cases (48.1%) of male breast cancer and in 147 cases (55.3%) of female breast cancer (p = 0.178). Further, expression rate of CD44v6 was significantly lower (p<0.001) and of CAXII significantly higher (p = 0.001) in male compared to female breast cancer. However, expression rates of the hypoxia markers CAIX and GLUT1 were not significantly different between male and female breast cancer (p = 0.865 and p = 0.164, respectively) (Table 2).

We found that co-expression of growth factor receptors was equally frequent in male (18.0%) and female (20.7%) breast cancer (p = 0.178). As shown in Table 3, co-expression of IGF1-R with other growth factor receptors was comparable between male (15.7%) and female breast cancer (13.6%) (p = 0.187). Furthermore, co-expression of EGFR with HER2 was similar in male (2.3%) and female (3.0%) breast cancer (p = 0.665), while co-expression rates for MET with EGFR and MET with FGFR2 were higher in female than in male breast cancer (4.5% vs. 0.0%, p = 0.013) and (5.6% vs. 0.8%, p = 0.019), respectively. Simultaneous expression of more than 3 growth factor receptors was found in 2.3% of male and 4.1% of female breast cancer cases (p = 0.336).

**Table 3:** Co-expression of growth factor receptors in male and female breast cancer.

	Co-expression with:			
Growth factor receptor	FGFR2 N (%)	MET N (%)	EGFR N (%)	HER2 N (%)
IGF1-R				
male	8 (6.0)	3 (2.3)	8 (6.0)	2 (1.5)
female	13 (4.9)	13 (4.9)	6 (2.3)	5 (1.9)
HER2				
male	1 (0.8)	1 (0.8)	3 (2.3)	
female	5 (1.8)	8 (3.0)	8 (3.0)	
EGFR				
male	3 (2.3)	0 (0.0)		
female	3 (1.1)	12 (4.5)		
MET				
male	1 (0.8)			
female	15 (5.6)			

### Clinicopathological Correlations of Membrane Marker Expression

Like in female breast cancer, expression of HER2 in male breast cancer correlated with high histological grade (p = 0.023) and tumor-size (p<0.001). In contrast to female breast cancer, expression of EGFR, GLUT1, and CAIX in male breast cancer did not correlate with any clinicopathological feature. Expression of IGF1-R, MET, FGFR2, and CD44v6 was not correlated to clinicopathological features in both male and female breast cancer. Nevertheless, expression of HER2, MET, EGFR, GLUT1, and CAIX was, as expected, significantly associated with loss of ERα and PR expression in female breast cancer (p<0.001, p = 0.027, p<0.001, p<0.001, p<0.001 respectively), whereas FGFR2 and CAXII were associated with ERα and PR expression (p = 0.003 and p = 0.016; Table 4). In male breast cancer, only expression of EGFR was significantly associated with loss of ERα and PR expression, probably due to a limited number of ERα and PR negative male breast cancer cases.

**Table 4:** Membrane marker expression in relation to hormone receptor expression.

	Male			Female		
Feature	ERα/PR positive	ERα/PR negative		ERα/PR positive	ERα/PR negative	
	N (%)	N (%)	p-value	N (%)	N (%)	p-value
IGF1-R						
Negative	76 (60.8)	4 (80.0)	0.387	177 (83.5)	42 (89.4)	0.314
Positive	49 (39.2)	1 (20.0)		35 (16.5)	5 (10.6)	
HER2						
Negative	124 (96.9)	5 (100.0)	0.688	207 (95.0)	39 (81.3)	**<0.001**
Positive	4 (3.1)	0 (0.0)		11 (5.0)	9 (18.7)	
EGFR						
Negative	114 (89.8)	3 (60.0)	**0.040**	208 (96.3)	18 (37.5)	**<0.001**
Positive	13 (10.2)	2 (40.0)		8 (3.7)	30 (62.5)	
MET						
Negative	121 (95.3)	5 (100.0)	0.619	175 (80.6)	31 (66.0)	**0.027**
Positive	6 (4.7)	0 (0.0)		42 (19.4)	16 (34.0)	
FGFR2						
Negative	111 (87.4)	5 (100.0)	0.397	152 (72.7)	43 (93.5)	**0.003**
Positive	16 (12.6)	0 (0.0)		57 (27.3)	3 (6.5)	
GLUT1						
Negative	85 (67.5)	5 (100.0)		155 (79.1)	19 (43.2)	
Positive	41 (32.5)	0 (0.0)	0.124	41 (20.9)	25 (56.8)	**<0.001**
CAIX						
Negative	118 (92.9)	5 (100.0)		189 (95.5)	25 (56.8)	
Positive	9 (7.1)	0 (0.0)	0.537	9 (4.5)	19 (43.2)	**<0.001**
CAXII						
Negative	92 (71.9)	5 (100.0)		190 (88.8)	47 (100.0)	
Positive	36 (28.1)	0 (0.0)	0.165	24 (11.2)	0 (0.0)	**0.016**
CD44v6						
Negative	83 (65.4)	5 (100.0)		77 (35.8)	16 (33.3)	
Positive	44 (34.6)	0 (0.0)	0.107	138 (64.2)	32 (66.7)	0.745

### Towards a Panel of Potential Membrane Markers for Molecular Imaging

**Figure 2:**
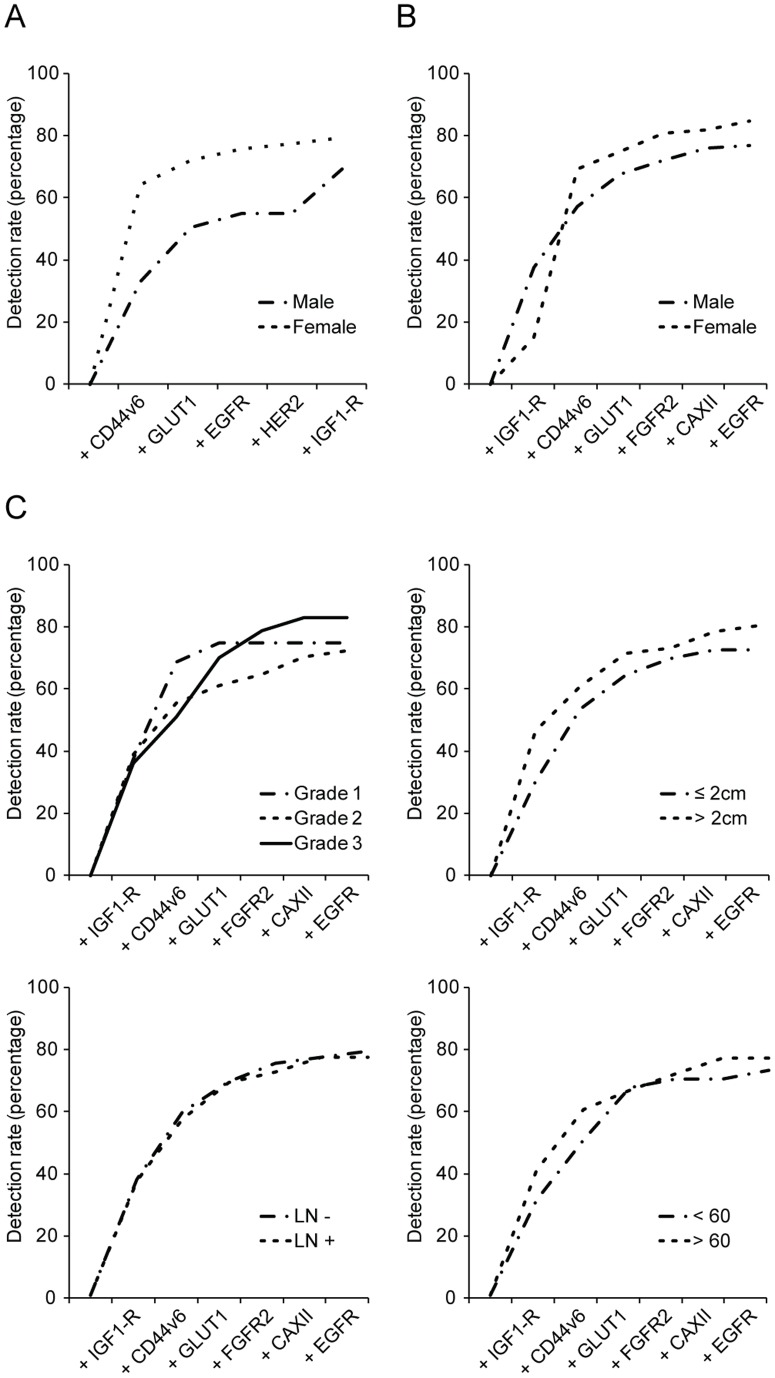
Potential detection rate of a panel of membrane markers for molecular imaging. Detection rate was calculated by the fraction of positive cases over the total population, taking into account that cancers can express multiple markers. A. Detection rate of the previously described panel in male and female breast cancer. B. The optimal combination of markers to detect male breast cancer. C. The detection rate of the panel in relation to clinicopathological features of male breast cancer.

From the previous results it became clear that all growth factor receptors are too infrequently expressed in male breast cancer to serve as individual targets for molecular imaging strategies, meaning that a panel of growth factor receptors supplemented with membrane-bound tumor markers is required to obtain a sufficient detection rate like in female breast cancer [24]. Our previously proposed panel consisting of CD44v6, EGFR, HER2, IGF1-R, and GLUT1 for imaging of female breast cancer was significantly less sensitive for imaging of male breast cancer (female breast cancer 79.3% vs. male breast cancer 69.1%, p = 0.025; Figure 2A). Inclusion of FGFR2 increased the difference in detection rate between male and female breast cancer (detection rate of female breast cancer 85.0% vs. male breast cancer 72.9%, p = 0.004). However, inclusion of CAXII resulted in the optimal detection rate possible for male breast cancer (76.7%, Figure 2B). Because the panel of membrane markers mainly consists of growth factor receptors and hypoxia markers, we investigated whether high grade male breast cancers are more frequently detected than low grade cancers. The sensitivity of a combination of growth factor receptors, supplemented with hypoxia markers, and CD44v6 was independent of histological grade, tumor size, lymph node status, or age (Figure 2C).

### Specificity of a Panel of Potential Membrane Markers in Male Breast Cancer

Since male breast cancer is often diagnosed together with gynecomastia, although gynecomastia is not an obligate precursor of male breast cancer [28], we examined whether the expression of the selected membrane markers was specific for male breast cancer. We found that expression patterns in gynecomastia were largely comparable with normal female breast tissue; i.e. no expression of HER2, EGFR, MET, GLUT1 and CAIX detectable. However, we observed FGFR2 expression in 3 cases (9.4%), IGF1-R expression in 1 case (3.1%), and a clear membranous staining of CAXII in 2 cases (6.3%). Gynecomastia was positive for CD44v6 (predominant staining of the myoepithelium) in all cases like normal female breast epithelium, which is probably not influencing the sensitivity of detection as we previously stated [24]. No difference in expression between the subtypes of gynecomastia was observed.

In summary, the expression of growth factor receptors in male and female breast cancer differs. However for molecular imaging strategies, the most optimal panel of potential membrane proteins for imaging of male breast cancer was similar to female breast cancer. Therefore a panel composed of IGF-1R, CD44v6, GLUT1, CAXII, FGFR2, and EGFR might be suitable for molecular imaging of male breast cancer.

## Discussion

The aim of this study was to identify the expression patterns of growth factor receptors in male breast cancer, and to determine whether growth factor receptors are suitable candidates for imaging strategies in male breast cancer patients. In addition, these markers could be potential candidates for targeted therapy in the near future next to hormonal therapy using tamoxifen and aromatase inhibitors. In order to determine the expression patterns we stained tissue microarrays containing 133 clinical specimens of male breast cancer by immunohistochemistry and compared it with 266 clinical specimens of female breast cancers and 32 cases of gynecomastia.

We found that expression of IGF1-R was present in 38.5%, FGFR2 in 12.1%, EGFR in 11.4%, HER2 in 3.0%, and MET in 4.5% of male breast cancers. Compared to female breast cancer, IGF1-R expression was higher and MET and FGFR2 expression lower in male breast cancer. In total, half of male breast cancers expressed one of the selected receptors. Furthermore, we found that 18.0% of male breast cancer patients expressed more than one growth factor receptor. The most predominant combination was IGF1-R with EGFR or FGFR2 expression.

The expression rates of HER2 and EGFR in male breast cancer were comparable with recent findings [5]–[7], [10], but other data for IGF1-R and MET in the male breast cancer literature are unavailable. Since MET expression is more prevalent in non-luminal female breast cancer, it seemed likely that MET expression in male breast cancer would be low (4.5%), because only 6% of male breast cancers was non-luminal type. IGF1-R expression was found in almost 40% of male breast cancers, suggesting that IGF1-R might be the driving growth factor receptor in male breast cancer. As described by the study of Peyrat et al [32] and Stoll [33], IGF1-R expression is related to ERα positivity, since almost all male breast cancers are ERα positive, high IGF1-R expression was expected. Further, FGFR2 expression in female breast cancer was highly correlated with ERα, PR expression and low grade [34], and with cancers in patients with a BRCA2 mutation [35]. Given that BRCA2 mutations are more frequent in male breast cancer [36], [37], FGFR2 levels were expected to be higher in male than in female breast cancer. However, in our study population FGFR2 expression in male breast cancer was two-fold lower than in female breast cancer.

When only growth factor receptors are used for molecular imaging, the sensitivity would be low (48.1%), however the specificity (as compared to gynecomastia) would be high. Due to low sensitivity, expression patterns of hypoxia markers and CD44v6 were studied in male breast cancer. We found that the ERα and hypoxia associated marker CAXII was more frequently expressed in male compared to female breast cancer. This could not be explained by ERα expression or a different regulation of the hypoxia response, because expression of CAIX and GLUT1 was not significantly different in male and female breast cancer [38], [39]. Therefore, gender specific differences e.g. hormonal balances probably play a role. Finally, we found that the expression of CD44v6 was significantly lower in male breast cancer than in female breast cancer, although no differential expression was seen between normal female breast tissue and gynecomastia, as judged by extensive staining of the myoepithelial cells. The underlying mechanism and clinical consequences of low CD44v6 in male breast cancer remains to be elucidated.

In conclusion, we found that expression of individual growth factor receptors (IGF1-R, FGFR2, and MET) and CD44v6, CAXII in male breast cancer is different compared to female breast cancer. However, when used as a panel of markers for molecular imaging strategies, the potential detection rate is similar for male and female breast cancer. This implies that membrane targets for molecular imaging of female breast cancer can also be used for detecting male breast cancer. The feasibility of molecular imaging (and therapy) of male breast cancer requires further study, but the present study thereby serves as a starting point for development of a set of antibody-based therapeutics and molecular tracers for male breast cancer.

## References

[1] Miao H, Verkooijen HM, Chia KS, Bouchardy C, Pukkala E, et al (2011) Incidence and outcome of male breast cancer: an international population-based study. J Clin Oncol 29: 4381–4386. 21969512 10.1200/JCO.2011.36.8902

[2] Anderson WF, Jatoi I, Tse J, Rosenberg PS (2010) Male breast cancer: a population-based comparison with female breast cancer. J Clin Oncol 28: 232–239. 19996029 10.1200/JCO.2009.23.8162 PMC2815713

[3] Fentiman IS, Fourquet A, Hortobagyi GN (2006) Male breast cancer. Lancet 367: 595–604. 16488803 10.1016/S0140-6736(06)68226-3

[4] Giordano SH, Cohen DS, Buzdar AU, Perkins G, Hortobagyi GN (2004) Breast carcinoma in men: a population-based study. Cancer 101: 51–57. 15221988 10.1002/cncr.20312

[5] Shaaban AM, Ball GR, Brannan RA, Cserni G, Di Benedetto A, et al (2012) A comparative biomarker study of 514 matched cases of male and female breast cancer reveals gender-specific biological differences. Breast Cancer Res Treat 133: 949–958. 22094935 10.1007/s10549-011-1856-9

[6] Ge Y, Sneige N, Eltorky MA, Wang Z, Lin E, et al (2009) Immunohistochemical characterization of subtypes of male breast carcinoma. Breast Cancer Res 11: R28. 19442295 10.1186/bcr2258 PMC2716496

[7] Kornegoor R, Verschuur-Maes AH, Buerger H, Hogenes MC, de Bruin PC, et al (2012) Molecular subtyping of male breast cancer by immunohistochemistry. Mod Pathol 25: 398–404. 22056953 10.1038/modpathol.2011.174

[8] Ciocca V, Bombonati A, Gatalica Z, Di Pasquale M, Milos A, et al (2006) Cytokeratin profiles of male breast cancers. Histopathology 49: 365–370. 16978199 10.1111/j.1365-2559.2006.02519.x

[9] Moore J, Friedman MI, Gansler T, Gramlich TL, Derose PB, et al (1998) Prognostic indicators in male breast carcinoma. Breast J 4: 261–269. 21223446 10.1046/j.1524-4741.1998.440261.x

[10] Foerster R, Foerster FG, Wulff V, Schubotz B, Baaske D, et al (2011) Matched-pair analysis of patients with female and male breast cancer: a comparative analysis. BMC Cancer 11: 335. 21816051 10.1186/1471-2407-11-335 PMC3199869

[11] Willsher PC, Leach IH, Ellis IO, Bell JA, Elston CW, et al (1997) Male breast cancer: pathological and immunohistochemical features. Anticancer Res 17: 2335–2338. 9245247

[12] Fox SB, Rogers S, Day CA, Underwood JC (1992) Oestrogen receptor and epidermal growth factor receptor expression in male breast carcinoma. J Pathol 166: 13–18. 1538271 10.1002/path.1711660104

[13] Pivot X, Bedairia N, Thiery-Vuillemin A, Espie M, Marty M (2011) Combining molecular targeted therapies: clinical experience. Anticancer Drugs 22: 701–710. 21467916 10.1097/CAD.0b013e328345ffa4

[14] Valachis A, Mauri D, Polyzos NP, Chlouverakis G, Mavroudis D, et al (2011) Trastuzumab combined to neoadjuvant chemotherapy in patients with HER2-positive breast cancer: a systematic review and meta-analysis. Breast 20: 485–490. 21784637 10.1016/j.breast.2011.06.009

[15] Garrett CR, Eng C (2011) Cetuximab in the treatment of patients with colorectal cancer. Expert Opin Biol Ther 11: 937–949. 21557708 10.1517/14712598.2011.582464

[16] Brockstein BE (2011) Management of recurrent head and neck cancer: recent progress and future directions. Drugs 71: 1551–1559. 21861540 10.2165/11592540-000000000-00000

[17] Sampath L, Kwon S, Ke S, Wang W, Schiff R, et al (2007) Dual-labeled trastuzumab-based imaging agent for the detection of human epidermal growth factor receptor 2 overexpression in breast cancer. J Nucl Med 48: 1501–1510. 17785729 10.2967/jnumed.107.042234

[18] Lee SB, Hassan M, Fisher R, Chertov O, Chernomordik V, et al (2008) Affibody molecules for in vivo characterization of HER2-positive tumors by near-infrared imaging. Clin Cancer Res 14: 3840–3849. 18559604 10.1158/1078-0432.CCR-07-4076 PMC3398736

[19] Gee MS, Upadhyay R, Bergquist H, Alencar H, Reynolds F, et al (2008) Human breast cancer tumor models: molecular imaging of drug susceptibility and dosing during HER2/neu-targeted therapy. Radiology 248: 925–935. 18647846 10.1148/radiol.2482071496 PMC2798096

[20] Pleijhuis RG, Graafland M, de Vries J, Bart J, de Jong JS, et al (2009) Obtaining adequate surgical margins in breast-conserving therapy for patients with early-stage breast cancer: current modalities and future directions. Ann Surg Oncol 16: 2717–2730. 19609829 10.1245/s10434-009-0609-z PMC2749177

[21] Pleijhuis RG, Langhout GC, Helfrich W, Themelis G, Sarantopoulos A, et al (2011) Near-infrared fluorescence (NIRF) imaging in breast-conserving surgery: assessing intraoperative techniques in tissue-simulating breast phantoms. Eur J Surg Oncol 37: 32–39. 21106329 10.1016/j.ejso.2010.10.006

[22] Hirche C, Murawa D, Mohr Z, Kneif S, Hunerbein M (2010) ICG fluorescence-guided sentinel node biopsy for axillary nodal staging in breast cancer. Breast Cancer Res Treat 121: 373–378. 20140704 10.1007/s10549-010-0760-z

[23] Crane LM, Themelis G, Arts HJ, Buddingh KT, Brouwers AH, et al (2011) Intraoperative near-infrared fluorescence imaging for sentinel lymph node detection in vulvar cancer: first clinical results. Gynecol Oncol 120: 291–295. 21056907 10.1016/j.ygyno.2010.10.009

[24] Vermeulen JF, van Brussel AS, van der Groep P, Morsink FH, Bult P, et al (2012) Immunophenotyping invasive breast cancer: paving the road for molecular imaging. BMC Cancer 12: 240. 22695343 10.1186/1471-2407-12-240 PMC3430576

[25] Vermeulen JF, van de Ven RA, Ercan C, van der Groep P, van der Wall E, et al (2012) Nuclear Kaiso expression is associated with high grade and triple-negative invasive breast cancer. PLoS One 7: e37864. 22662240 10.1371/journal.pone.0037864 PMC3360634

[26] Elston CW, Ellis IO (1991) Pathological prognostic factors in breast cancer. I. The value of histological grade in breast cancer: experience from a large study with long-term follow-up. Histopathology 19: 403–410. 1757079 10.1111/j.1365-2559.1991.tb00229.x

[27] van der Groep P, Bouter A, van der Zanden R, Siccama I, Menko FH, et al (2006) Distinction between hereditary and sporadic breast cancer on the basis of clinicopathological data. J Clin Pathol 59: 611–617. 16603649 10.1136/jcp.2005.032151 PMC1860390

[28] Kornegoor R, Verschuur-Maes AH, Buerger H, van Diest PJ (2012) The 3-layered ductal epithelium in gynecomastia. Am J Surg Pathol 36: 762–768. 22314184 10.1097/PAS.0b013e31824324e6

[29] CCMO website: Central Committee on Research involving Human Subjects (Centrale Commissie Mensgebonden Onderzoek) (text in Dutch). Available: http://www.ccmo-online.nl/main.asp?pid=10&sid=30&ssid=51. Accessed: 2012 Oct 1.

[30] van Diest PJ (2002) No consent should be needed for using leftover body material for scientific purposes. For. BMJ 325: 648–651. 12242180

[31] Kornegoor R, Verschuur-Maes AH, Buerger H, Hogenes MC, de Bruin PC, et al (2012) Fibrotic focus and hypoxia in male breast cancer. Mod Pathol 25: 1397–1404. 22684218 10.1038/modpathol.2012.101

[32] Peyrat JP, Bonneterre J, Beuscart R, Djiane J, Demaille A (1988) Insulin-like growth factor 1 receptors in human breast cancer and their relation to estradiol and progesterone receptors. Cancer Res 48: 6429–6433. 2972358

[33] Stoll BA (2002) Oestrogen/insulin-like growth factor-I receptor interaction in early breast cancer: clinical implications. Ann Oncol 13: 191–196. 11885994 10.1093/annonc/mdf059

[34] Garcia-Closas M, Hall P, Nevanlinna H, Pooley K, Morrison J, et al (2008) Heterogeneity of breast cancer associations with five susceptibility loci by clinical and pathological characteristics. PLoS Genet 4: e1000054. 18437204 10.1371/journal.pgen.1000054 PMC2291027

[35] Bane AL, Pinnaduwage D, Colby S, Reedijk M, Egan SE, et al (2009) Expression profiling of familial breast cancers demonstrates higher expression of FGFR2 in BRCA2-associated tumors. Breast Cancer Res Treat 117: 183–191. 18563556 10.1007/s10549-008-0087-1 PMC2727582

[36] Gethins M (2012) Breast cancer in men. J Natl Cancer Inst 104: 436–438. 22393212 10.1093/jnci/djs172

[37] Evans DG, Susnerwala I, Dawson J, Woodward E, Maher ER, et al (2010) Risk of breast cancer in male BRCA2 carriers. J Med Genet 47: 710–711. 20587410 10.1136/jmg.2009.075176

[38] Barnett DH, Sheng S, Charn TH, Waheed A, Sly WS, et al (2008) Estrogen receptor regulation of carbonic anhydrase XII through a distal enhancer in breast cancer. Cancer Res 68: 3505–3515. 18451179 10.1158/0008-5472.CAN-07-6151

[39] Wykoff CC, Beasley N, Watson PH, Campo L, Chia SK, et al (2001) Expression of the hypoxia-inducible and tumor-associated carbonic anhydrases in ductal carcinoma in situ of the breast. Am J Pathol 158: 1011–1019. 11238049 10.1016/S0002-9440(10)64048-5 PMC1850356

